# Supplementary figures and images for: Pooled analysis of oral microbiome profiles defines robust signatures associated with periodontitis
Source: mSystems. 2024 Oct 24;9(11):e00930-24. doi: 10.1128/msystems.00930-24 (PMC11575188; doi:10.1128/msystems.00930-24)

A

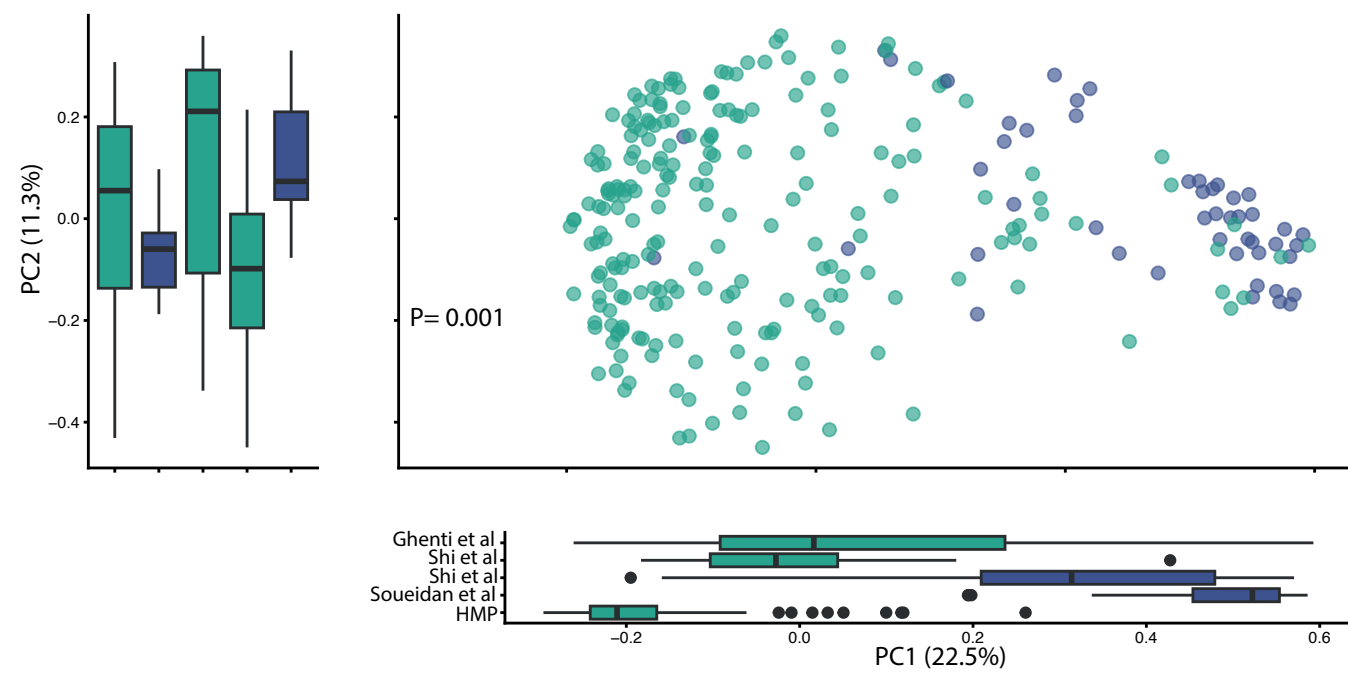

B

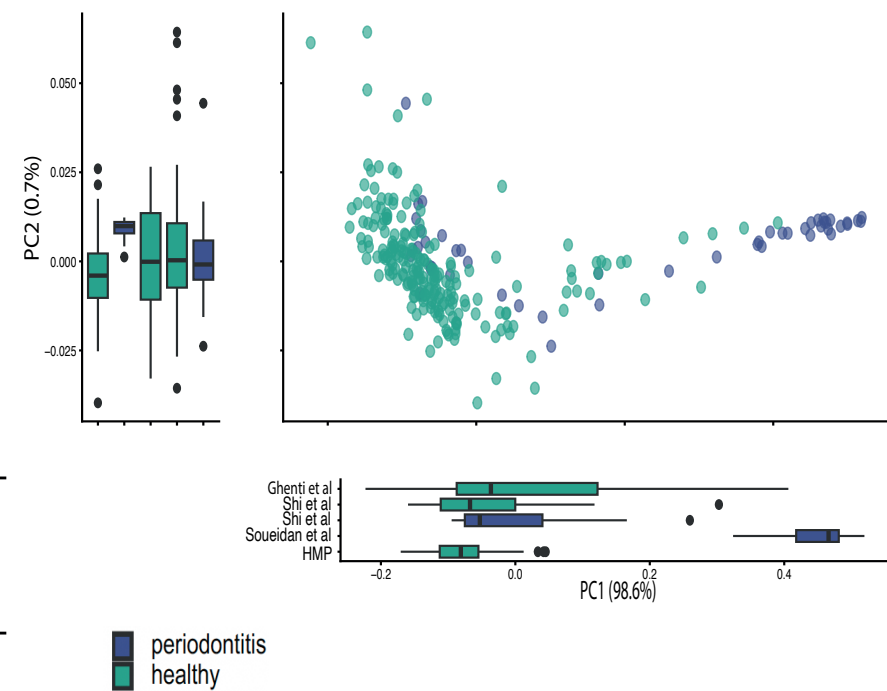

C

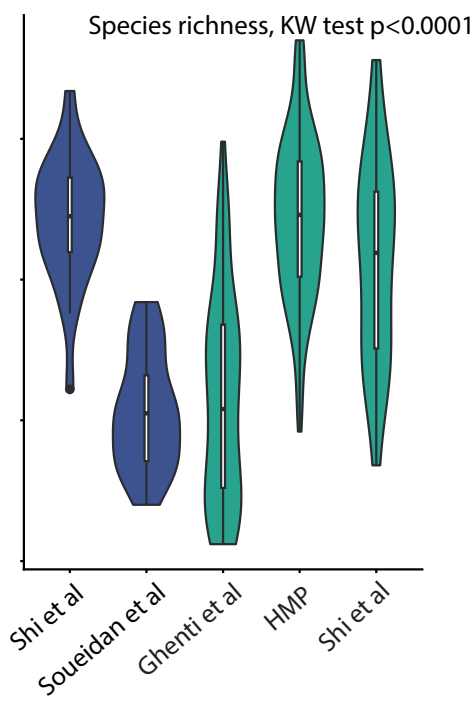

D

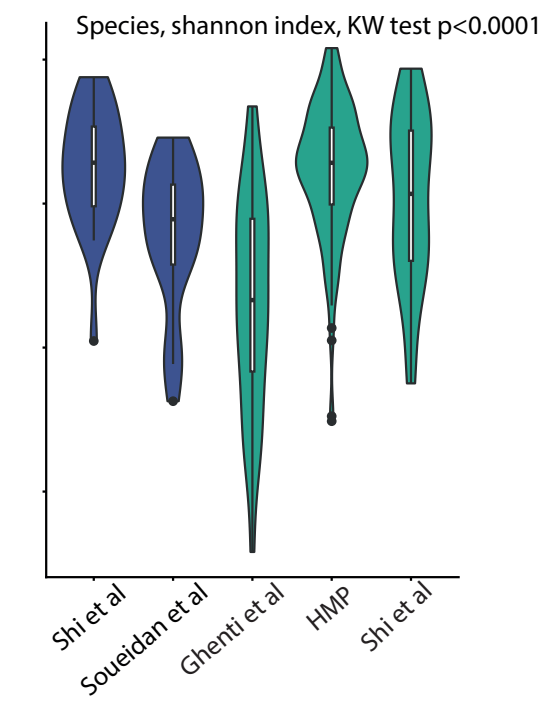

E

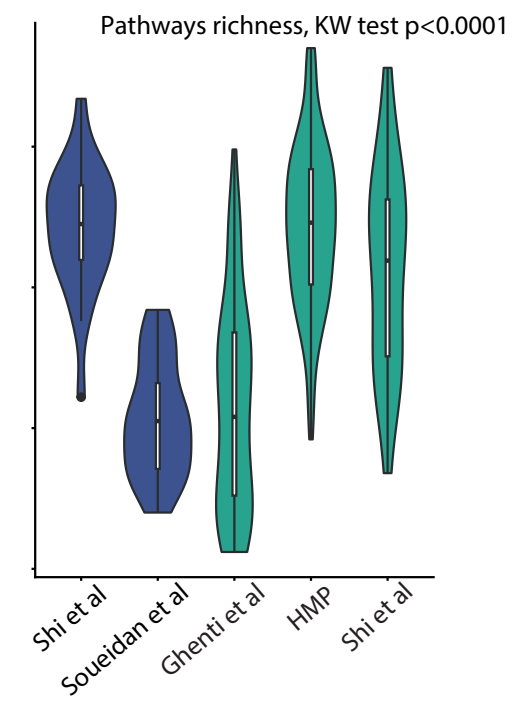

F

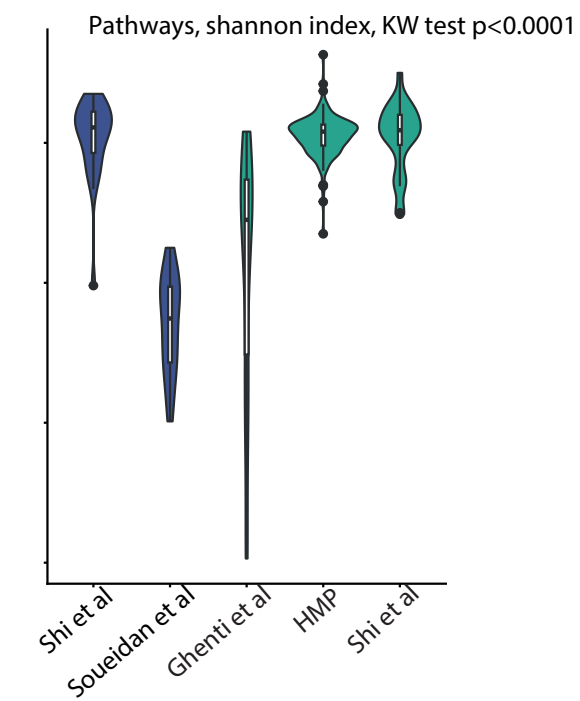

Supplement: Figure S1 — Principal-coordinate analyses and alpha diversity determinations. [file msystems.00930-24-s0001.pdf]

A

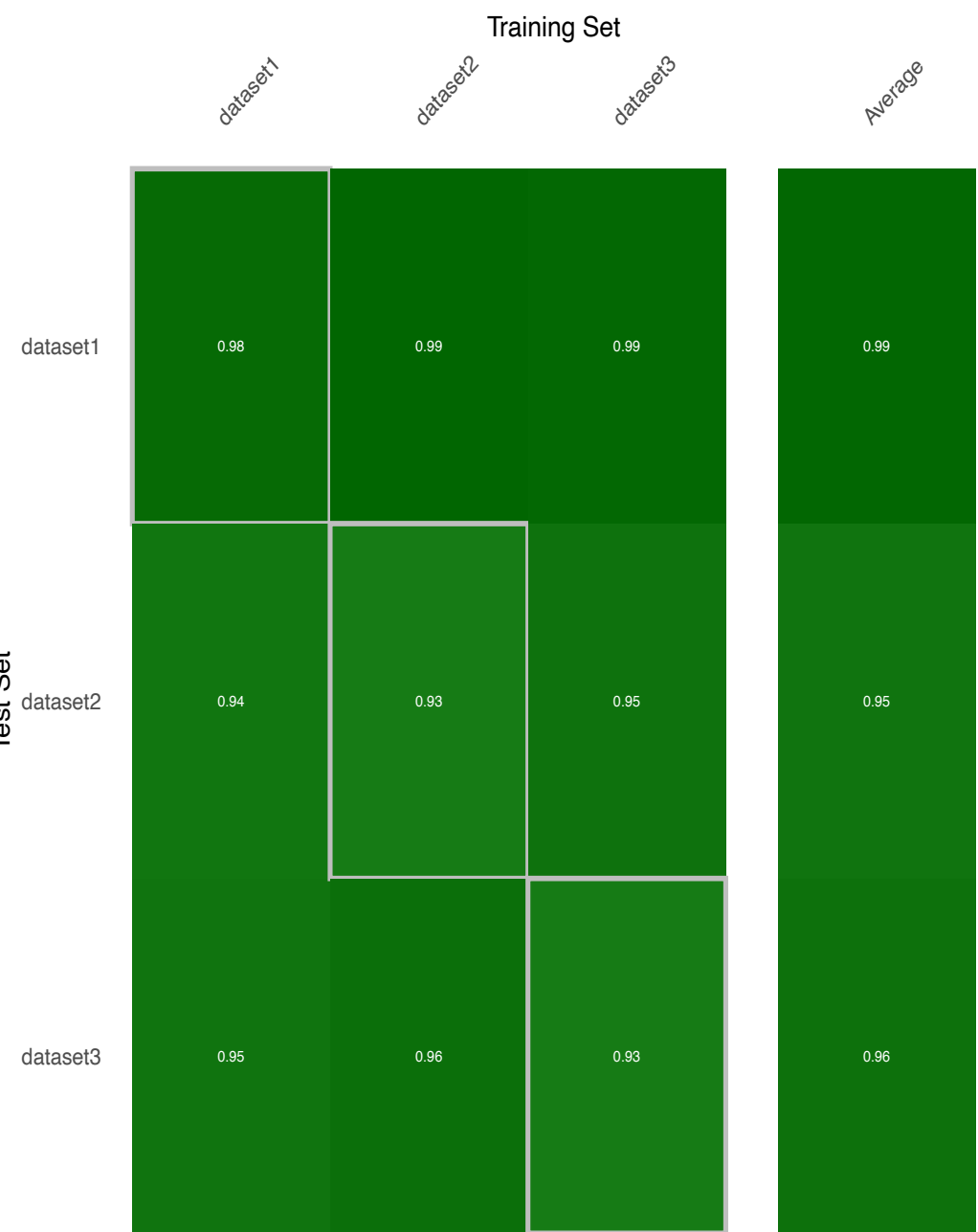

B

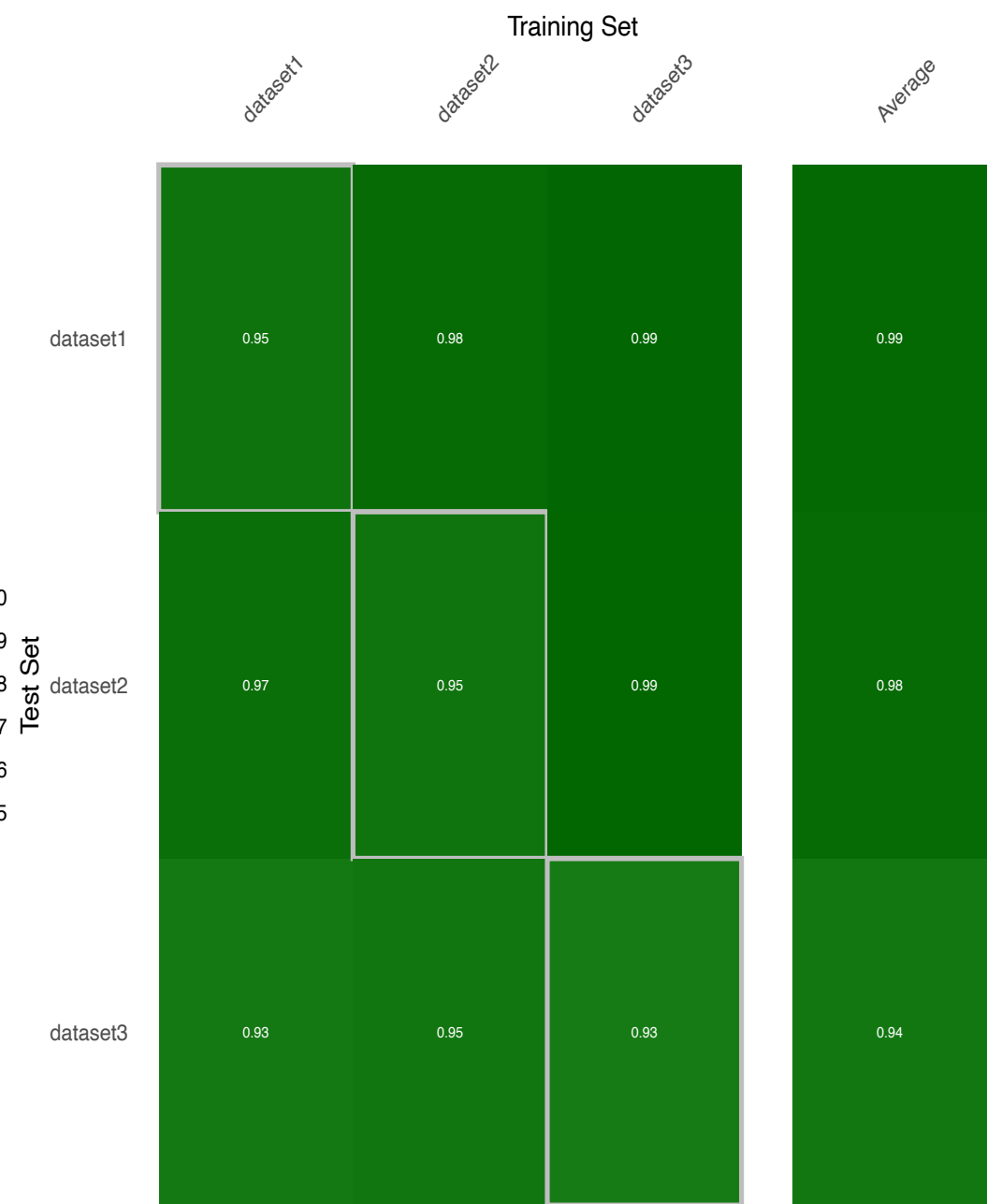

Supplement: Figure S3 — Prediction matrix. [file msystems.00930-24-s0003.pdf]
