# Supplementary material for: Pooled analysis of oral microbiome profiles defines robust signatures associated with periodontitis
Source: mSystems. 2024 Oct 24;9(11):e00930-24. doi: 10.1128/msystems.00930-24 (PMC11575188; doi:10.1128/msystems.00930-24)
Supplement: Figure S2 — Most common dominant bacteria in the oral microbiome and triad of the Socransky's red complex. [file msystems.00930-24-s0002.pdf]

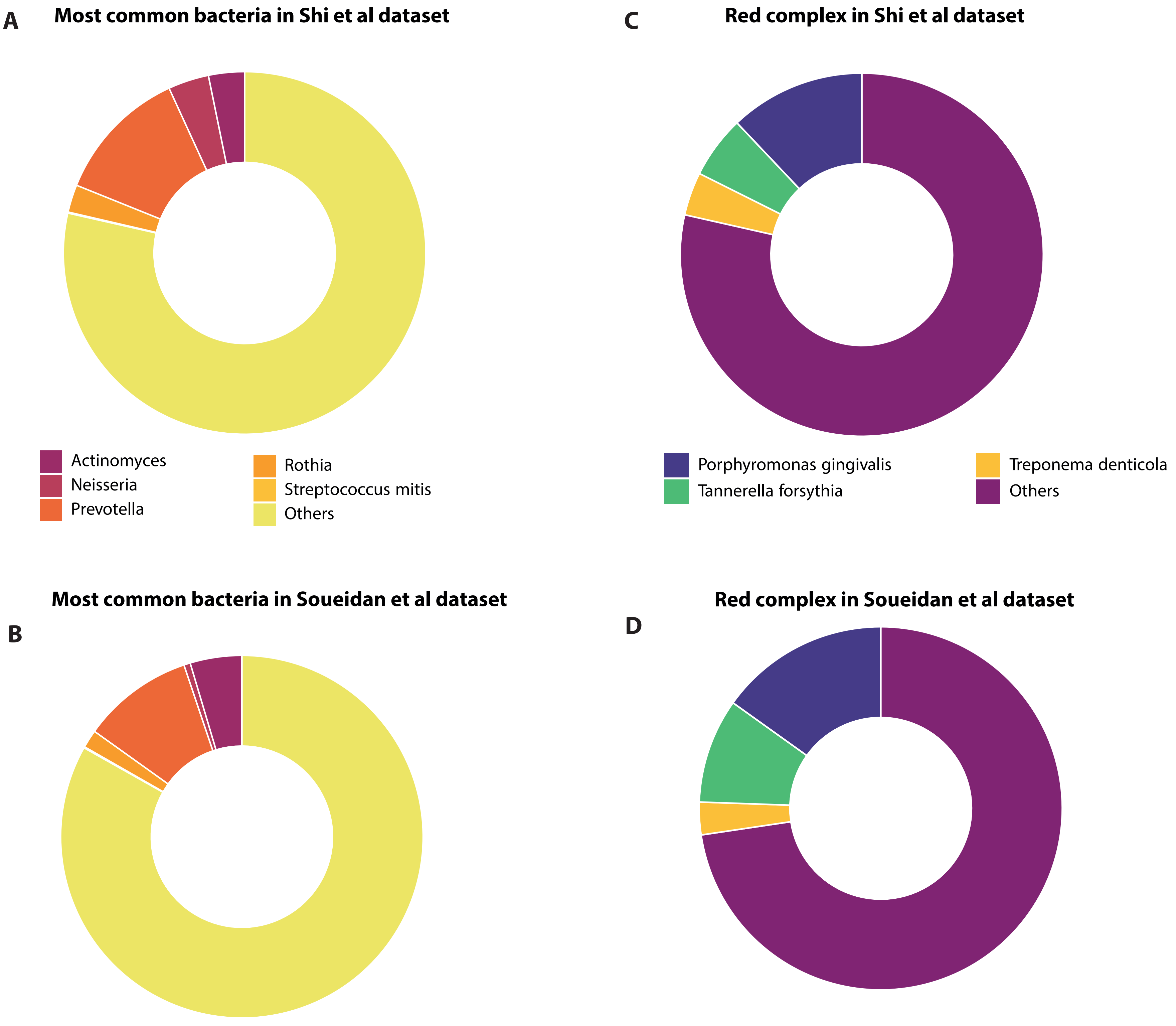
A
Most common bacteria in Shi et al dataset
Actinomyces
Neisseria
Prevotella
Rothia
Streptococcus mitis
Others
C
Red complex in Shi et al dataset
Porphyromonas gingivalis
Tannerella forsythia
Treponema denticola
Others
B
Most common bacteria in Soueidan et al dataset
D
Red complex in Soueidan et al dataset
