# Supplementary material for: Pooled analysis of oral microbiome profiles defines robust signatures associated with periodontitis
Source: mSystems. 2024 Oct 24;9(11):e00930-24. doi: 10.1128/msystems.00930-24 (PMC11575188; doi:10.1128/msystems.00930-24)
Supplement: Figure S4 — Fast-and-frugal tree-based staging schemes to predict periodontitis at species level. [file msystems.00930-24-s0004.pdf]

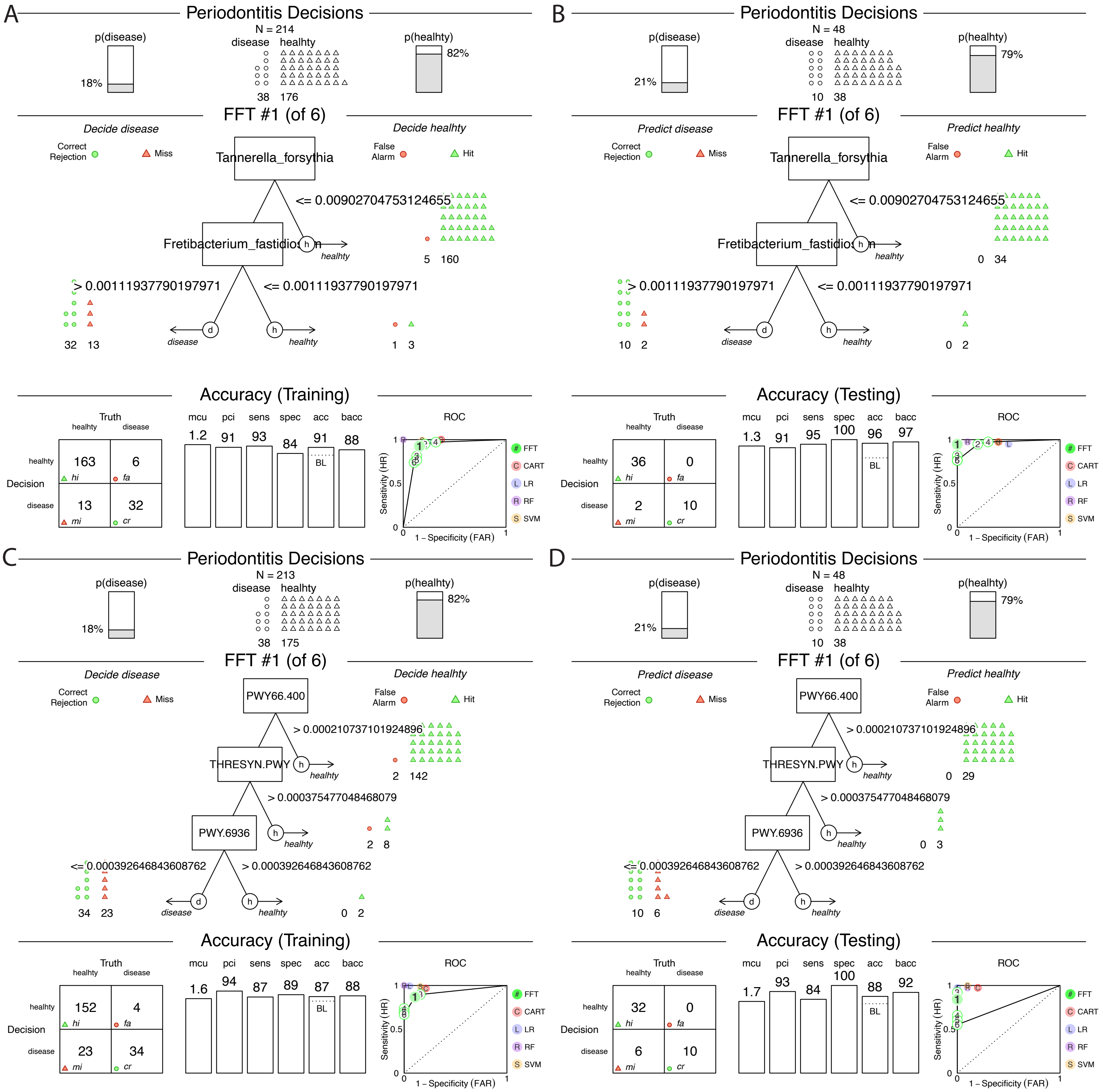

A
Periodontitis Decisions
N = 214
p(disease)
18%
disease
healhty
38
176
p(healhty)
82%
FFT #1 (of 6)
Decide disease
Correct Rejection
Miss
Decide healhty
False Alarm
Hit
Tannerella_forsythia
<= 0.00902704753124655
5
160
Fretibacterium_fastidiosum
healhty
> 0.00111937790197971
<= 0.00111937790197971
disease
healhty
32
13
1
3
Accuracy (Training)
Truth
healhty
disease
Decision
healhty
163
hi
6
fa
disease
13
mi
32
cr
mcu 1.2
pci 91
sens 93
spec 84
acc 91
bacc 88
BL
ROC
Sensitivity (HR)
1 – Specificity (FAR)
FFT
CART
LR
RF
SVM
B
Periodontitis Decisions
N = 48
p(disease)
21%
disease
healhty
10
38
p(healhty)
79%
FFT #1 (of 6)
Predict disease
Correct Rejection
Miss
Predict healhty
False Alarm
Hit
Tannerella_forsythia
<= 0.00902704753124655
0
34
Fretibacterium_fastidiosum
healhty
> 0.00111937790197971
<= 0.00111937790197971
disease
healhty
10
2
0
2
Accuracy (Testing)
Truth
healhty
disease
Decision
healhty
36
hi
0
fa
disease
2
mi
10
cr
mcu 1.3
pci 91
sens 95
spec 100
acc 96
bacc 97
BL
ROC
Sensitivity (HR)
1 – Specificity (FAR)
FFT
CART
LR
RF
SVM
C
Periodontitis Decisions
N = 213
p(disease)
18%
disease
healhty
38
175
p(healhty)
82%
FFT #1 (of 6)
Decide disease
Correct Rejection
Miss
Decide healhty
False Alarm
Hit
PWY66.400
> 0.000210737101924896
2
142
THRESYN.PWY
healhty
> 0.000375477048468079
2
8
PWY.6936
healhty
<= 0.000392646843608762
> 0.000392646843608762
disease
healhty
34
23
0
2
Accuracy (Training)
Truth
healhty
disease
Decision
healhty
152
hi
4
fa
disease
23
mi
34
cr
mcu 1.6
pci 94
sens 87
spec 89
acc 87
bacc 88
BL
ROC
Sensitivity (HR)
1 – Specificity (FAR)
FFT
CART
LR
RF
SVM
D
Periodontitis Decisions
N = 48
p(disease)
21%
disease
healhty
10
38
p(healhty)
79%
FFT #1 (of 6)
Predict disease
Correct Rejection
Miss
Predict healhty
False Alarm
Hit
PWY66.400
> 0.000210737101924896
0
29
THRESYN.PWY
healhty
> 0.000375477048468079
0
3
PWY.6936
healhty
<= 0.000392646843608762
> 0.000392646843608762
disease
healhty
10
6
Accuracy (Testing)
Truth
healhty
disease
Decision
healhty
32
hi
0
fa
disease
6
mi
10
cr
mcu 1.7
pci 93
sens 84
spec 100
acc 88
bacc 92
BL
ROC
Sensitivity (HR)
1 – Specificity (FAR)
FFT
CART
LR
RF
SVM
