# Supplementary material for: Pooled analysis of oral microbiome profiles defines robust signatures associated with periodontitis
Source: mSystems. 2024 Oct 24;9(11):e00930-24. doi: 10.1128/msystems.00930-24 (PMC11575188; doi:10.1128/msystems.00930-24)
Supplement: Supplemental methods — R custom codes used. [file msystems.00930-24-s0005.docx]

# ##### ALPHA DIVERSITY SHANNON SPECIES

x <- t(read.delim(species_table.txt', row.names = 1))

m <- read.delim('mapping_file.txt', row.names = 1)

dim(x)

rownames(x)

colnames(x)

rownames(m)

x <- sweep(x,1,rowSums(x),'/')

x <- x[rownames(m),]

rownames(m) == rownames(x)

#MWU adjusted on study

#STATUS (periodontis YES/ periodontis NO)

library(coin)

shannon <- as.data.frame(diversity(t(x), index = "shannon"))

wilcox_test(shannon $shannon ~ as.factor(m$STATUS)|as.factor(m$study)) kruskal_test(shannon $shannon ~as.factor(m$STATUS)|as.factor(m$study))

#limma

fit <- lmFit(as.numeric(SHANNON), mm)

fit <- eBayes(fit)

head(coef(fit))

limma_res_df <- data.frame(topTable(fit, coef = "statushealthy", number = Inf)) limma_res_df

library(FSA)

dunnTest(shannon $shannon ~ m$ study,

method="bonferroni")

pdf('alpha_div_ SHANNON.pdf',width=4,height=4);

e <- ggplot(SHANNON ,aes(x = STATUS, y = SHANNON))

e + geom_violin(aes(fill = STATUS)) +

geom_boxplot(width=0.05) +

scale_fill_manual(values = col) +

#scale_fill_jco()+

theme_cowplot(font_size = 7) +

theme(axis.text.x = element_text(color=NA), axis.text.y = element_text(color=NA))

dev.off()

# ###### ALPHA DIVERSITY OS

x <- t(read.delim(species_table.txt', row.names = 1))

m <- read.delim('mapping_file.txt', row.names = 1)

dim(x)

rownames(x)

colnames(x)

rownames(m)

x <- sweep(x,1,rowSums(x),'/')

x <- x[rownames(m),]

rownames(m) == rownames(x)

#MWU adjusted on study

library(coin)

OS <- as.data.frame(specnumber(x))

wilcox_test(OS$OS ~ as.factor(m$STATUS)|as.factor(m$study))

kruskal_test(OS$OS ~as.factor(m$STATUS)|as.factor(m$study))

#limma

fit <- lmFit(as.numeric(OS), mm)

fit <- eBayes(fit)

head(coef(fit))

limma_res_df <- data.frame(topTable(fit, coef = "statushealthy", number = Inf)) limma_res_df

library(FSA)

dunnTest(OS$OS ~ m$study,

method="bonferroni")

pdf('alpha_div_ OS.pdf',width=4,height=4);

e <- ggplot(OS ,aes(x = STATUS, y = OS))

e + geom_violin(aes(fill = STATUS)) +

geom_boxplot(width=0.05) +

scale_fill_manual(values = col) +

#scale_fill_jco()+

theme_cowplot(font_size = 7) +

theme(axis.text.x = element_text(color=NA), axis.text.y = element_text(color=NA))

dev.off()

# ##### ALPHA DIVERSITY SHANNON PATHWAYS

x <- t(read.delim(pathways_table.txt', row.names = 1))

m <- read.delim('mapping_file.txt', row.names = 1)

dim(x)

rownames(x)

colnames(x)

rownames(m)

x <- sweep(x,1,rowSums(x),'/')

x <- x[rownames(m),]

rownames(m) == rownames(x)

#MWU adjusted on study

library(coin)

shannon <- as.data.frame(diversity(t(x), index = "shannon"))

wilcox_test(shannon $shannon ~ as.factor(m$STATUS)|as.factor(m$study)) kruskal_test(shannon $shannon ~as.factor(m$STATUS)|as.factor(m$study))

#limma

fit <- lmFit(as.numeric(SHANNON), mm)

fit <- eBayes(fit)

head(coef(fit))

limma_res_df <- data.frame(topTable(fit, coef = "statushealthy", number = Inf)) limma_res_df

library(FSA)

dunnTest(shannon $shannon ~ m$ study,

method="bonferroni")

pdf('alpha_div_ SHANNON_PATH.pdf',width=4,height=4);

e <- ggplot(SHANNON ,aes(x = STATUS, y = SHANNON))

e + geom_violin(aes(fill = STATUS)) +

geom_boxplot(width=0.05) +

scale_fill_manual(values = col) +

#scale_fill_jco()+

theme_cowplot(font_size = 7) +

theme(axis.text.x = element_text(color=NA), axis.text.y = element_text(color=NA))

dev.off()

# ##### ALPHA DIVERSITY Observed Pathways

x <- t(read.delim(species_table.txt', row.names = 1))

m <- read.delim('mapping_file.txt', row.names = 1)

dim(x)

rownames(x)

colnames(x)

rownames(m)

x <- sweep(x,1,rowSums(x),'/')

x <- x[rownames(m),]

rownames(m) == rownames(x)

#MWU adjusted on study

library(coin)

OS <- as.data.frame(specnumber(x))

wilcox_test(OS$OS ~ as.factor(m$STATUS)|as.factor(m$study))

kruskal_test(OS$OS ~as.factor(m$STATUS)|as.factor(m$study))

#limma

fit <- lmFit(as.numeric(OS), mm)

fit <- eBayes(fit)

head(coef(fit))

limma_res_df <- data.frame(topTable(fit, coef = "statushealthy", number = Inf)) limma_res_df

library(FSA)

dunnTest(OS$OS ~ m$study,

method="bonferroni")

pdf('alpha_div_ OS_PATH.pdf',width=4,height=4);

e <- ggplot(OS ,aes(x = STATUS, y = OS))

e + geom_violin(aes(fill = STATUS)) +

geom_boxplot(width=0.05) +

scale_fill_manual(values = col) +

#scale_fill_jco()+

theme_cowplot(font_size = 7) +

theme(axis.text.x = element_text(color=NA), axis.text.y = element_text(color=NA))

dev.off()

# ############### BETA DIVERSITY SPECIES ###################

x <- t(read.delim('table_speciestxt', row.names = 1))

m <- read.delim('mapping_file.txt', row.names = 1)

dim(x)

rownames(x)

colnames(x)

rownames(m)

x <- sweep(x,1,rowSums(x),'/')

x <- x[rownames(m),]

rownames(m) == rownames(x)

dim(x)

beta_table <- as.matrix(vegdist(x), method = "bray", na.rm = F)

PCOA <- pcoa(beta_table)$vectors

dim(PCOA)

dim(m)

var_exp <- pcoa(beta_table)$values

rownames(m)

map2 <- m

colnames(map2)

rownames(PCOA) == rownames(map2)

#Run stats for diff. centroids

beta_dist = as.dist(beta_table)

length(beta_dist)

#Run STATUS (periodontis YES/ periodontis NO)

ad = adonis2(beta_dist ~ map2$STATUS, permutations=999)

ad

adonis2(formula = beta_dist ~ map2$STATUS + map2$study, permutations = 999)

kruskal_test(PCOA[,1] ~ as.factor(m$STATUS)|as.factor(map2$study))

kruskal_test(PCOA[,2] ~ as.factor(m$STATUS)|as.factor(map2$study))

col = viridis(10)[c(3,6)]

for(i in 1:ncol(PCOA)){

colnames(PCOA)[i] <- paste("PC",i, sep="")

}

PCOA <- cbind(PCOA, rownames(PCOA))

colnames(PCOA)[ncol(PCOA)] <- "SampleID"

mapping2 <- map2

mapping2 <- cbind(mapping2, rownames(PCOA))

mapping2 <- data.frame(lapply(mapping2, as.character), stringsAsFactors=FALSE)

colnames(mapping2)[ncol(mapping2)] <- "SampleID"

PCOA <- merge(PCOA, mapping2, by="SampleID")

as.numeric(as.character(PCOA$PC1))

PCOA$PC1 <- as.numeric(as.character(PCOA$PC1))

PCOA$PC2 <- as.numeric(as.character(PCOA$PC2))

PCOA$PC3 <- as.numeric(as.character(PCOA$PC3))

PCOA$PC4 <- as.numeric(as.character(PCOA$PC4))

body_PCOA <- ggplot(PCOA) +

geom_point(size = 2, alpha=0.65, aes_string(x = "PC1", y = "PC2", color = "STATUS" )) +

#scale_fill_jco()

scale_color_manual(values=col) +

#scale_color_jco() +

scale_fill_manual(values = col) +

theme_cowplot(font_size = 7) +

guides(color=F) +

annotate("text", x=-0.45, y=-0.2, label= paste("P=", p_val), size=2) +

annotate("text", x=-0.45, y=-0.25, label= paste("R2=", round(r_sq, digits=3)), size=2) +

#guides(color=guide_legend(nrow=3)) +

labs(x="", y="") +

theme(axis.text.x = element_text(color=NA), axis.text.y = element_text(color=NA))

#Make boxplot of PCs

PC1_boxes <- ggplot(PCOA) +

geom_boxplot(aes_string(x = factor(PCOA$STATUS, levels=c("diseased", "healhty")), y = "PC1", fill = "STATUS")) +

#scale_fill_jco() +

#scale_fill_viridis(discrete = TRUE, option = "D") +

scale_fill_manual(values = col) +

theme_cowplot(font_size = 7) +

guides(fill=F)+

coord_flip() +

labs(x="", y= paste("PC1 (", round(var_exp$Relative_eig[1], digits=3)*100, "%)", sep=""))

PC2_boxes <- ggplot(PCOA) +

geom_boxplot(aes_string(x =factor(PCOA$STATUS, levels=c("diseased", "healhty")), y = "PC2", fill = "STATUS")) +

#scale_fill_jco() +

#scale_fill_viridis(discrete = TRUE, option = "D") +

scale_fill_manual(values = col) +

theme_cowplot(font_size = 7) +

guides(fill=F) +

labs(x ="", y= paste("PC2 (", round(var_exp$Relative_eig[2], digits=3)*100, "%)", sep="")) +

theme(axis.text.x = element_text(color=NA))

#Compile the PCoA and boxes

top2 <- plot_grid(PC2_boxes, body_PCOA, ncol=2, rel_widths=c(0.3, 1))

bottom2 <- plot_grid(NULL, PC1_boxes, ncol=2, rel_widths=c(0.3, 1))

together2 <- plot_grid(top2, bottom2, nrow=2, rel_heights=c(1, 0.3))

pdf('betadiv_SPECIES.pdf',width=7,height=3.5);

together2

dev.off()

# ############### BETA DIVERSITY PATHWAYS ###################

x <- t(read.delim('table_speciestxt', row.names = 1))

m <- read.delim('mapping_file.txt', row.names = 1)

dim(x)

rownames(x)

colnames(x)

rownames(m)

x <- sweep(x,1,rowSums(x),'/')

x <- x[rownames(m),]

rownames(m) == rownames(x)

dim(x)

beta_table <- as.matrix(vegdist(x), method = "bray", na.rm = F)

PCOA <- pcoa(beta_table)$vectors

dim(PCOA)

dim(m)

var_exp <- pcoa(beta_table)$values

rownames(m)

map2 <- m

colnames(map2)

rownames(PCOA) == rownames(map2)

#Run stats for diff. centroids

beta_dist = as.dist(beta_table)

length(beta_dist)

#Run STATUS (periodontis YES/ periodontis NO)

ad = adonis2(beta_dist ~ map2$STATUS, permutations=999)

ad

adonis2(formula = beta_dist ~ map2$STATUS + map2$study, permutations = 999)

kruskal_test(PCOA[,1] ~ as.factor(m$STATUS)|as.factor(map2$study))

kruskal_test(PCOA[,2] ~ as.factor(m$STATUS)|as.factor(map2$study))

col = viridis(10)[c(3,6)]

for(i in 1:ncol(PCOA)){

colnames(PCOA)[i] <- paste("PC",i, sep="")

}

PCOA <- cbind(PCOA, rownames(PCOA))

colnames(PCOA)[ncol(PCOA)] <- "SampleID"

mapping2 <- map2

mapping2 <- cbind(mapping2, rownames(PCOA))

mapping2 <- data.frame(lapply(mapping2, as.character), stringsAsFactors=FALSE)

colnames(mapping2)[ncol(mapping2)] <- "SampleID"

PCOA <- merge(PCOA, mapping2, by="SampleID")

as.numeric(as.character(PCOA$PC1))

PCOA$PC1 <- as.numeric(as.character(PCOA$PC1))

PCOA$PC2 <- as.numeric(as.character(PCOA$PC2))

PCOA$PC3 <- as.numeric(as.character(PCOA$PC3))

PCOA$PC4 <- as.numeric(as.character(PCOA$PC4))

body_PCOA <- ggplot(PCOA) +

geom_point(size = 2, alpha=0.65, aes_string(x = "PC1", y = "PC2", color = "STATUS" )) +

#scale_fill_jco()

scale_color_manual(values=col) +

#scale_color_jco() +

scale_fill_manual(values = col) +

theme_cowplot(font_size = 7) +

guides(color=F) +

annotate("text", x=-0.45, y=-0.2, label= paste("P=", p_val), size=2) +

annotate("text", x=-0.45, y=-0.25, label= paste("R2=", round(r_sq, digits=3)), size=2) +

#guides(color=guide_legend(nrow=3)) +

labs(x="", y="") +

theme(axis.text.x = element_text(color=NA), axis.text.y = element_text(color=NA))

#Make boxplot of PCs

PC1_boxes <- ggplot(PCOA) +

geom_boxplot(aes_string(x = factor(PCOA$STATUS, levels=c("diseased", "healhty")), y = "PC1", fill = "STATUS")) +

#scale_fill_jco() +

#scale_fill_viridis(discrete = TRUE, option = "D") +

scale_fill_manual(values = col) +

theme_cowplot(font_size = 7) +

guides(fill=F)+

coord_flip() +

labs(x="", y= paste("PC1 (", round(var_exp$Relative_eig[1], digits=3)*100, "%)", sep=""))

PC2_boxes <- ggplot(PCOA) +

geom_boxplot(aes_string(x =factor(PCOA$STATUS, levels=c("diseased", "healhty")), y = "PC2", fill = "STATUS")) +

#scale_fill_jco() +

#scale_fill_viridis(discrete = TRUE, option = "D") +

scale_fill_manual(values = col) +

theme_cowplot(font_size = 7) +

guides(fill=F) +

labs(x ="", y= paste("PC2 (", round(var_exp$Relative_eig[2], digits=3)*100, "%)", sep="")) +

theme(axis.text.x = element_text(color=NA))

#Compile the PCoA and boxes

top2 <- plot_grid(PC2_boxes, body_PCOA, ncol=2, rel_widths=c(0.3, 1))

bottom2 <- plot_grid(NULL, PC1_boxes, ncol=2, rel_widths=c(0.3, 1))

together2 <- plot_grid(top2, bottom2, nrow=2, rel_heights=c(1, 0.3))

pdf('betadiv_PATH.pdf',width=7,height=3.5);

together2

dev.off()

# ########### MOST COMMON SPECIES ##############

x <- t(read.delim('table_SPECIES.txt', row.names = 1))

m <- read.delim('mapping_file.txt', row.names = 1)

x <- sweep(x,1,rowSums(x),'/')

x <- x[rownames(m),]

rownames(m) == rownames(x)

#healthy individuals #######

m$STATUS

meta<- subset(m , STATUS == "healthy")

x_ARDS<- as.data.frame(x[(rownames(meta_ICU)),])

#Streptococcus mitis, Prevotella, Actinomyces, Neisseria, and Rothia

grep("Streptococcus_mitis", colnames(x_ARDS_ICU))

colnames(x_ARDS_ICU)[732]

grep("Prevotella", colnames(x_ARDS_ICU))

c(582:635)

grep("Actinomyces", colnames(x_ARDS_ICU))

c(15:44)

grep("Neisseria", colnames(x_ARDS_ICU))

c(501:518)

grep("Rothia", colnames(x_ARDS_ICU))

c(663:665)

count.data <- data.frame(

class = c("Streptococcus_mitis", "Prevotella", "Actinomyces", "Neisseria","Rothia", "others" ),

prop = c(mean((x_ARDS_ICU)[,717])*100, mean((Prevotella)[,1])*100, mean((Actinomyces)[,1])*100, mean((Neisseria)[,1])*100, mean((Rothia)[,1])*100,

100-mean((x_ARDS_ICU)[,717])*100 - mean((Prevotella)[,1])*100 - mean((Actinomyces)[,1])*100- mean((Neisseria)[,1])*100-mean((Rothia)[,1])*100)

)

count.data

count.data <- count.data %>%

arrange(desc(class)) %>%

mutate(lab.ypos = cumsum(prop) - 0.5*prop)

count.data

pdf('most_common_bacteria_healhty.pdf',width=6,height=6);

ggplot(count.data, aes(x = 2, y = prop, fill = class)) +

geom_bar(width = 1, stat = "identity", color = "white") +

coord_polar("y", start = 0)+

scale_fill_manual(values = c("#9F2A63FF", "#BB3754FF", "#E8602DFF" , "#FB9E07FF", "#FAC127FF", "#F3E55CFF" )) +

theme_void() + xlim(0.5, 2.5) + ggtitle("Most common bacteria in healthy subjects")+ theme(plot.title = element_text(hjust = 0.5, face="bold"))

dev.off()

#diseased ############

meta<- subset(m , STATUS == "diseased")

x_ARDS<- x[(rownames(meta_ICU)),]

#Streptococcus mitis, Prevotella, Actinomyces, Neisseria, and Rothia

grep("Streptococcus_mitis", colnames(x_ARDS_ICU))

colnames(x_ARDS_ICU)[717]

grep("Prevotella", colnames(x_ARDS_ICU))

c(571:623)

grep("Actinomyces", colnames(x_ARDS_ICU))

c(14:43)

grep("Neisseria", colnames(x_ARDS_ICU))

c(492:509)

grep("Rothia", colnames(x_ARDS_ICU))

c(650:652)

count.data <- data.frame(

class = c("Streptococcus_mitis", "Prevotella", "Actinomyces", "Neisseria","Rothia", "Zothers" ),

prop = c(mean((x_ARDS_ICU)[,717])*100, mean((Prevotella)[,1])*100, mean((Actinomyces)[,1])*100, mean((Neisseria)[,1])*100, mean((Rothia)[,1])*100,

100-mean((x_ARDS_ICU)[,717])*100 - mean((Prevotella)[,1])*100 - mean((Actinomyces)[,1])*100- mean((Neisseria)[,1])*100-mean((Rothia)[,1])*100)

)

count.data

count.data <- count.data %>%

arrange(desc(class)) %>%

mutate(lab.ypos = cumsum(prop) - 0.5*prop)

count.data

pdf('most_common_bacteria_diseased.pdf',width=6,height=6);

ggplot(count.data, aes(x = 2, y = prop, fill = class)) +

geom_bar(width = 1, stat = "identity", color = "white") +

coord_polar("y", start = 0)+

scale_fill_manual(values = c("#9F2A63FF", "#BB3754FF", "#E8602DFF" , "#FB9E07FF", "#FAC127FF", "#F3E55CFF" )) +

theme_void() + xlim(0.5, 2.5) + ggtitle("Most common bacteria in diseased samples")+ theme(plot.title = element_text(hjust = 0.5, face="bold"))

dev.off()

# DIFFERENTIAL SPECIES

phyloseqin <- t(read.delim('table_SPECIES.txt', row.names = 1))

rownames(x)

colSums(x)

rowSums(x)

metadata <- read.delim('mapping_file.txt', row.names = 1)

metadatadf <- data.frame(metadata)

row.names(metadatadf) <- metadatadf$sample_id

samples_df <- metadatadf %>% select (-sample_id)

sample <- sample_data(samples_df)

phyloseqin <- subset_taxa(phyloseqin, !is.na(Species))

phyloseqin

taxa_names(phyloseqin) <- gsub("s__", "", taxa_names(phyloseqin)) #removing s__ in OTU names

sample_variables(phyloseqin)

sample_data(phyloseqin)$STATUS <- factor(sample_data(phyloseqin)$STATUS, levels = c("diseased", "healthy"))

table(sample_data(phyloseqin)$STATUS)

#1) CORN_DA

corn_da <- differentialTest(formula = ~ STATUS + study ,

phi.formula = ~ STATUS,

formula_null = ~ study ,

phi.formula_null = ~ 1,

data = phyloseqin,

test = "Wald", boot = FALSE,

fdr_cutoff = 0.05)

plot(corn_da, level = "Species")

fdr_corncob <- corn_da$significant_taxa

#2) Limma-Voom with TMM

dds <- phyloseq_to_deseq2(phyloseqin, ~ STATUS) dge <- as.DGEList(dds)

dge <- calcNormFactors(dge, method = "TMM")

mm <- model.matrix(~ 0 + STATUS + study , dge$samples)

head(mm)

table(mm[, 2])

y <- voom(dge, mm, plot = T)

fit <- lmFit(y, mm)

fit <- eBayes(fit)

head(coef(fit))

limma_res_df <- data.frame(topTable(fit, coef = "groupdiseased", number = Inf))

fdr_limma <- limma_res_df %>%

dplyr::filter(adj.P.Val < 0.05) %>%

rownames_to_column(var = "Species")

#3) DESeq2 with apeglm

dds <- phyloseq_to_deseq2(phyloseqin, design = ~ STATUS + study)

dds <- DESeq(dds, test = "Wald", fitType = "local", sfType = "poscounts")

plotDispEsts(dds)

res <- lfcShrink(dds, coef=2, type="apeglm")

deseq_res_df <- data.frame(res) %>%

rownames_to_column(var = "Species") %>%

dplyr::arrange(padj)

fdr_deseq <- deseq_res_df %>%

dplyr::filter(padj < 0.05)

#4) MAASLIN2

mas_1 <- Maaslin2(

input_data = data.frame(otu_table(phyloseqin)),

input_metadata = data.frame(sample_data(phyloseqin)),

min_abundance = 0.0,

min_prevalence = 0.0,

normalization = "TSS",

transform = "LOG",

analysis_method = "LM",

max_significance = 0.05,

fixed_effects = c("STATUS","study"),

correction = "BH",

output = "/Maaslin_OUT ",

standardize = FALSE,

reference = c("study "),

cores = 1)

mas_res_df <- mas_1$results

fdr_mas <- mas_res_df %>%

dplyr::filter(qval < 0.05)

#5) ANCOM

ancom_da <- ancombc(phyloseq = phyloseqin, formula = "STATUS + study",

p_adj_method = "fdr", zero_cut = 0.90, lib_cut = 1000,

group = "STATUS", struc_zero = TRUE, neg_lb = TRUE, tol = 1e-5,

max_iter = 100, conserve = TRUE, alpha = 0.05, global = FALSE)

ancom_res_df <- data.frame(

Species = row.names(ancom_da$res$beta),

beta = unlist(ancom_da$res$beta),

se = unlist(ancom_da$res$se),

W = unlist(ancom_da$res$W),

p_val = unlist(ancom_da$res$p_val),

q_val = unlist(ancom_da$res$q_val),

diff_abn = unlist(ancom_da$res$diff_abn))

fdr_ancom <- ancom_res_df %>%

dplyr::filter(q_val < 0.05)

dim(fdr_ancom)

# ########### PLOT DIFFERENTIAL FEATURES

###########################################################################################################################################################################

#create label

label <- create.label(meta=metadatadf, label="STATUS",

case = "diseased", control="healthy")

###########################################################################################################################################################################

#create siamat object

sc.obj <- siamcat(feat=feat.all_dif_PATH,

label=label,

meta=as.data.frame(metadatadf))

show(sc.obj)

###########################################################################################################################################################################

#filter

sc.obj <- filter.features(sc.obj,

filter.method = 'abundance',

cutoff = 0.001)

sc.obj <- filter.features(sc.obj, cutoff=0.05,

filter.method='prevalence',

feature.type = 'filtered')

body_cols <- viridis(10)[c(6,3)]

sc.obj <- check.associations(sc.obj, detect.lim = 1e-06,

alpha=0.10, max.show = 20,

plot.type = 'quantile.rect',

panels = c('fc'),

fn.plot = './association_plot_species.pdf', color.scheme = body_cols)

############################################################################################################################################

########### build the roc curve #############################################################################################################

##############################################################################################################################################

#test the model

#model building

sc.obj <- normalize.features(

sc.obj,

norm.method = "log.unit",

norm.param = list(

log.n0 = 1e-06,

n.p = 2,

norm.margin = 1

)

)

sc.obj <- create.data.split(

sc.obj,

num.folds = 10,

num.resample = 10

)

sc.obj <- train.model(

sc.obj,

method = "lasso"

)

model_type(sc.obj)

models <- models(sc.obj)

models[[1]]

sc.obj <- make.predictions(sc.obj)

pred_matrix <- pred_matrix(sc.obj)

sc.obj <- evaluate.predictions(sc.obj)

model.evaluation.plot(sc.obj, fn.plot = './eval_plot_species.pdf', colours ="#800000FF")

# ##################### FAST AND FRUGAL TREES

library(FFTrees)

tree <- FFTrees(formula = STATUS ~ .,

data = train,

data.test = test,

main = "Periodontitis Decisions",

decision.labels = c("disease", "healhty"))

tree

inwords(tree)

tree$criterion_name

tree$cue_names

tree$formula

tree$data

tree$params

tree$competition

tree$cues

# Plot

pdf('FFTree_species.pdf',width=7,height=7);

plot(tree)

dev.off()

#plot(tree, tree=2)

pdf('FFTree_species_TEST.pdf',width=7,height=7);

plot(tree, data = 'test')

dev.off()

# ##### RANDOM SELECTION OF 3 DATASETS

x <- t(read.delim('table_species.txt', row.names = 1))

rownames(x)

colSums(x)

rowSums(x)

m <- read.delim('metadata.txt', row.names = 1)

a <- rownames(m)

b <-rownames(x)

x <- x[rownames(m),]

#create les fichier omni et nonomni

m_omni <- subset(m, STATUS =="healthy")

m_nonomni <- subset(m, STATUS =="diseased")

#split omni

library(semTools)

splitMyData_omni <- splitSample(m_omni, div = 3, type = "txt",

name = "splitSample")

map1_omni <- as.data.frame(splitMyData_omni[[1]])

map2_omni <- as.data.frame(splitMyData_omni[[2]])

map3_omni <- as.data.frame(splitMyData_omni[[3]])

#split nonomni

splitMyData_nonomni <- splitSample(m_nonomni, div = 3, type = "txt",

name = "splitSample")

map1_nonomni <- as.data.frame(splitMyData_nonomni[[1]])

map2_nonomni <- as.data.frame(splitMyData_nonomni[[2]])

map3_nonomni <- as.data.frame(splitMyData_nonomni[[3]])

#combined les mapping

map_1 <- as.data.frame(rbind(map1_nonomni, map1_omni))

map_1$STATUS

dim(map_1)

map_2 <- as.data.frame(rbind(map2_nonomni, map2_omni))

map_3 <- as.data.frame(rbind(map3_nonomni, map3_omni))

#add the number of the dataset last column

map_1 <- cbind(map_1, rep("dataset1", nrow(map_1)))

colnames(map_1)[6] <- c("dataset_number")

map_2 <- cbind(map_2, rep("dataset2", nrow(map_2)))

colnames(map_2)[6] <- c("dataset_number")

map_3 <- cbind(map_3, rep("dataset3", nrow(map_3)))

colnames(map_3)[6] <- c("dataset_number")

#split les tables taxonomie

x_1 <- x[rownames(map_1),]

x_2 <- x[rownames(map_2),]

x_3 <- x[rownames(map_3),]

#combine les mapping

map_combined <- as.data.frame(rbind(map_1, map_2, map_3))

dim(map_combined)

write.table(map_combined, paste("map_combined_metanalysis.txt"), sep='\t', quote = F, row.names = T, col.names = T)

#combine les table taxo

x_combined <- as.data.frame(rbind(x_1, x_2, x_3))

dim(x_combined)

rownames(x_combined) == rownames(map_combined)

write.table(t(x_combined), paste("x_combined_metanalysis.txt"), sep='\t', quote = F, row.names = T, col.names = T)

# ####META-ANALYSIS

datasets <- c('dataset1', 'dataset2', 'dataset3')

feat.all <- t(x_combined)

###########################################################################################################################################################################

#create label

map_combined$STATUS

label <- create.label(meta=map_combined, label="STATUS",

case = "diseased", control="healthy")

###########################################################################################################################################################################

#create siamat object

sc.obj <- siamcat(feat=feat.all,

label=label,

meta=map_combined)

show(sc.obj)

###########################################################################################################################################################################

#filter

sc.obj <- filter.features(sc.obj,

filter.method = 'abundance',

cutoff = 0.001)

sc.obj <- filter.features(sc.obj, cutoff=0.05,

filter.method='prevalence',

feature.type = 'filtered')

for (d in datasets){

# filter metadata and convert to dataframe

meta.train <- map_combined %>%

filter(dataset_number==d) %>%

as.data.frame()

rownames(meta.train) <-rownames(meta.train)

# create SIAMCAT object

sc.obj <- siamcat(feat=feat.all, meta=meta.train, label='STATUS', case='diseased')

# test for associations

sc.obj <- check.associations(sc.obj, alpha =0.05,

mult.corr = "fdr",

detect.lim = 10 ^-4, color.scheme = col,

feature.type = 'original',fn.plot = paste0('assoc_plot', d, '.pdf'))

# extract the associations and save them in the assoc.list

temp <- associations(sc.obj)

temp$species <- rownames(temp)

assoc.list[[d]] <- temp %>%

select(species, fc, auc, p.adj) %>%

mutate(Study=d)

}

# combine all associations

df.assoc <- bind_rows(assoc.list)

df.assoc <- df.assoc %>% filter(grepl('s__', species))

head(df.assoc)

strsplit(df.assoc$species, 's__')[2]

df.assoc$species <- as.character(map(strsplit(df.assoc$species, split = 's__'), 2))

genera.of.interest <- df.assoc %>%

group_by(species) %>%

summarise(m=mean(auc), n.filt=any(auc < 0.25 | auc > 0.75),

.groups='keep') %>%

filter(n.filt) %>%

arrange(m)

pdf('species_of_interest.pdf',width=7,height=7);

df.assoc %>%

# take only genera of interest

filter(species %in% genera.of.interest$species) %>%

# convert to factor to enforce an ordering by mean AUC

mutate(species=factor(species, levels = rev(genera.of.interest$species))) %>%

# convert to factor to enforce ordering again

mutate(Study=factor(Study, levels = datasets)) %>%

# annotate the cells in the heatmap with stars

mutate(l=case_when(p.adj < 10 ^-4~'*', TRUE~'')) %>%

ggplot(aes(y=species, x=Study, fill=fc)) +

geom_tile() +

scale_fill_gradient2(low = '#3B6FB6', high='#D41645', mid = 'white',

limits=c(-2.7, 2.7), name='Generalized\nfold change') +

theme_minimal() +

geom_text(aes(label=l)) +

theme(panel.grid = element_blank()) +

xlab('') + ylab('') +

theme(axis.text = element_text(size=6))

dev.off()

species_of_interest <- df.assoc %>%

# take only genera of interest

filter(species %in% genera.of.interest$species) %>%

# convert to factor to enforce an ordering by mean AUC

mutate(species=factor(species, levels = rev(genera.of.interest$species))) %>%

# convert to factor to enforce ordering again

mutate(Study=factor(Study, levels = datasets)) %>%

# annotate the cells in the heatmap with stars

mutate(l=case_when(p.adj < 0.05~'*', TRUE~''))

write.table(species_of_interest, paste("species_of_interest_ALL_soueidan.txt"), sep='\t', quote = F, row.names = T, col.names = T)

# create tibble to store all the predictions

auroc.all <- tibble(study.train=character(0),

study.test=character(0),

AUC=double(0))

# and a list to save the trained SIAMCAT objects

sc.list <- list()

for (i in datasets){

# restrict to a single study

meta.train <- map_combined %>%

filter(dataset_number==i) %>%

as.data.frame()

rownames(meta.train) <- rownames(meta.train)

# create SIAMCAT object

sc.obj.train <- siamcat(feat=feat.all, meta=meta.train,

label='STATUS', case='diseased')

# normalize features

sc.obj.train <- normalize.features(sc.obj.train, norm.method = 'log.std',

norm.param=list(log.n0=1e-05, sd.min.q=0),feature.type = 'original')

# Create data split

sc.obj.train <- create.data.split(sc.obj.train,

num.folds = 10, num.resample = 10)

# train LASSO model

sc.obj.train <- train.model(sc.obj.train, method='lasso')

## apply trained models to other datasets

# loop through datasets again

for (i2 in datasets){

if (i == i2){

# make and evaluate cross-validation predictions (same dataset)

sc.obj.train <- make.predictions(sc.obj.train)

sc.obj.train <- evaluate.predictions(sc.obj.train)

auroc.all <- auroc.all %>%

add_row(study.train=i, study.test=i,

AUC=eval_data(sc.obj.train)$auroc %>% as.double())

} else {

# make and evaluate on the external datasets

# use meta.ind here, since we want only one sample per subject!

meta.test <- map_combined %>%

filter(dataset_number==i2) %>%

as.data.frame()

rownames(meta.test) <- rownames(meta.test)

sc.obj.test <- siamcat(feat=feat.all, meta=meta.test,

label='STATUS', case='diseased')

# make holdout predictions

sc.obj.test <- make.predictions(sc.obj.train,

siamcat.holdout = sc.obj.test)

sc.obj.test <- evaluate.predictions(sc.obj.test)

auroc.all <- auroc.all %>%

add_row(study.train=i, study.test=i2,

AUC=eval_data(sc.obj.test)$auroc %>% as.double())

}

}

# save the trained model

sc.list[[i]] <- sc.obj.train

}

#After we trained and applied all models, we can calculate the test average for each dataset:

test.average <- auroc.all %>%

filter(study.train!=study.test) %>%

group_by(study.test) %>%

summarise(AUC=mean(AUC), .groups='drop') %>%

mutate(study.train="Average")

#Now that we have the AUROC values, we can plot them into a nice heatmap:

# combine AUROC values with test average

pdf('metaaalysis.pdf',width=7,height=7);

bind_rows(auroc.all, test.average) %>%

# highlight cross validation versus transfer results

mutate(CV=study.train == study.test) %>%

# for facetting later

mutate(split=case_when(study.train=='Average'~'Average', TRUE~'none')) %>%

mutate(split=factor(split, levels = c('none', 'Average'))) %>%

# convert to factor to enforce ordering

mutate(study.train=factor(study.train, levels=c(datasets, 'Average'))) %>%

mutate(study.test=factor(study.test, levels=c(rev(datasets),'Average'))) %>%

ggplot(aes(y=study.test, x=study.train, fill=AUC, size=CV, color=CV)) +

geom_tile() + theme_minimal() +

# text in tiles

geom_text(aes_string(label="format(AUC, digits=2)"),

col='white', size=2)+

# color scheme

scale_fill_gradientn(colours=rev(c('darkgreen','forestgreen',

'chartreuse3','lawngreen',

'yellow')), limits=c(0.5, 1)) +

# axis position/remove boxes/ticks/facet background/etc.

scale_x_discrete(position='top') +

theme(axis.line=element_blank(),

axis.ticks = element_blank(),

axis.text.x.top = element_text(angle=45, hjust=.1),

panel.grid=element_blank(),

panel.border=element_blank(),

strip.background = element_blank(),

strip.text = element_blank()) +

xlab('Training Set') + ylab('Test Set') +

scale_color_manual(values=c('#FFFFFF00', 'grey'), guide=FALSE) +

scale_size_manual(values=c(0, 1), guide=FALSE) +

facet_grid(~split, scales = 'free', space = 'free')

dev.off()

## Warning: It is deprecated to specify `guide = FALSE` to remove a guide. Please

## use `guide = "none"` instead.

## Warning: It is deprecated to specify `guide = FALSE` to remove a guide. Please

## use `guide = "none"` instead.

# Now that we the trained models (and we saved them in the sc.list object), we can also extract the model weights using SIAMCAT and compare to the associations we computed above.

weight.list <- list()

for (d in datasets){

sc.obj.train <- sc.list[[d]]

# extract the feature weights out of the SIAMCAT object

temp <- feature_weights(sc.obj.train)

temp$species <- rownames(temp)

# save selected info in the weight.list

weight.list[[d]] <- temp %>%

select(species, median.rel.weight, mean.rel.weight, percentage) %>%

mutate(Study=d) %>%

mutate(r.med=rank(-abs(median.rel.weight)),

r.mean=rank(-abs(mean.rel.weight)))

}

# combine all feature weights into a single tibble

df.weights <- bind_rows(weight.list)

df.weights <- df.weights %>% filter(grepl('s__', species))

head(df.weights)

strsplit(df.assoc$species, 's__')[2]

df.weights$species <- as.character(map(strsplit(df.weights$species, split = 's__'), 2))

#df.weights <- df.weights %>% filter(species!='unclassified')

# Using this, we can plot another heatmap with the weights, focussing on the genera of interest for which we plotted the associations as heatmap above.

# compute absolute feature weights

abs.weights <- df.weights %>%

group_by(Study) %>%

summarise(sum.median=sum(abs(median.rel.weight)),

sum.mean=sum(abs(mean.rel.weight)),

.groups='drop')

pdf('InvestigateFeatureWeights.pdf',width=7,height=7);

df.weights %>%

full_join(abs.weights) %>%

# normalize by the absolute model size

mutate(median.rel.weight=median.rel.weight/sum.median) %>%

# only include genera of interest

filter(species %in% genera.of.interest$species) %>%

# highlight feature rank for the top 20 features

mutate(r.med=case_when(r.med > 20~NA_real_, TRUE~r.med)) %>%

# enforce the correct ordering by converting to factors again

mutate(species=factor(species, levels = rev(genera.of.interest$species))) %>%

mutate(Study=factor(Study, levels = datasets)) %>%

ggplot(aes(y=species, x=Study, fill=median.rel.weight)) +

geom_tile() +

scale_fill_gradientn(colours=rev(

c('#007A53', '#009F4D', "#6CC24A", 'white',

"#EFC06E", "#FFA300", '#BE5400')),

limits=c(-0.15, 0.15)) +

theme_minimal() +

geom_text(aes(label=r.med), col='black', size= 2) +

theme(panel.grid = element_blank()) +

xlab('') + ylab('') +

theme(axis.text = element_text(size=6))

dev.off()

## Joining, by = "Study"

# ###################HOLD OUT procedure##################

################################### hold out dataset 1########

#convertir en phyloseq object

mphlanin <- t(read.csv("x_combined.txt", sep = "\t", strip.white = T, stringsAsFactors = F, row.names = 1))

mphlanin <- sweep(mphlanin,1,rowSums(mphlanin),'/')

metadata <- read.delim("map_combined.txt", header=TRUE, sep = "\t")

meta.fr <- subset(metadata, dataset_number != 'dataset1')

meta.fr$dataset_number

mphlanin_hold1234 <- mphlanin[rownames(meta.fr),]

feat.fr <- t(mphlanin_hold1234)

# create SIAMCAT object

siamcat.fr <- siamcat(feat=feat.fr, meta=meta.fr,

label='STATUS', case='diseased')

meta.cn <- subset(metadata, dataset_number == 'dataset1')

meta.cn$dataset_number

mphlanin_hold1 <- mphlanin[rownames(meta.cn),]

feat.cn <- t(mphlanin_hold1)

# SIAMCAT object

siamcat.cn <- siamcat(feat=feat.cn, meta=meta.cn,

label='STATUS', case='diseased')

siamcat.fr <- filter.features(

siamcat.fr,

filter.method = 'abundance',

cutoff = 0.001,

rm.unmapped = TRUE,

verbose=2

)

siamcat.fr <- normalize.features(

siamcat.fr,

norm.method = "log.std",

norm.param = list(log.n0 = 1e-06, sd.min.q = 0.1),

verbose = 2

)

siamcat.fr <- create.data.split(

siamcat.fr,

num.folds = 5,

num.resample = 2

)

siamcat.fr <- train.model(

siamcat.fr,

method = "lasso"

)

#prediction

siamcat.fr <- make.predictions(siamcat.fr)

## Made predictions successfully.

siamcat.fr <- evaluate.predictions(siamcat.fr)

## Evaluated predictions successfully.

#Application on the Holdout Dataset

siamcat.cn <- normalize.features(siamcat.cn,

norm.param=norm_params(siamcat.fr),

feature.type='original',

verbose = 2)

siamcat.cn <- make.predictions(

siamcat = siamcat.fr,

siamcat.holdout = siamcat.cn,

normalize.holdout = FALSE)

siamcat.cn <- evaluate.predictions(siamcat.cn)

#model evaluation

model.evaluation.plot('all_datasets_expect_one'=siamcat.fr,

'hold-out dataset 1'=siamcat.cn, fn.plot = './holdoutdataset1_redo.pdf', colours=c('#7E6148', '#91D1C2'))

# make the same for dataset 2 and 3

# ###############random forest PROCEDURE ##################

# create tibble to store all the predictions

auroc.all <- tibble(study.train=character(0),

study.test=character(0),

AUC=double(0))

# and a list to save the trained SIAMCAT objects

sc.list <- list()

for (i in datasets){

# restrict to a single study

meta.train <- map_combined %>%

filter(dataset_number==i) %>%

as.data.frame()

rownames(meta.train) <- rownames(meta.train)

# create SIAMCAT object

sc.obj.train <- siamcat(feat=feat.all, meta=meta.train,

label='STATUS', case='diseased')

# normalize features

sc.obj.train <- normalize.features(sc.obj.train, norm.method = 'log.std',

norm.param=list(log.n0=1e-05, sd.min.q=0),feature.type = 'original')

# Create data split

sc.obj.train <- create.data.split(sc.obj.train,

num.folds = 10, num.resample = 10)

# train LASSO model

sc.obj.train <- train.model(sc.obj.train, method='randomForest')

## apply trained models to other datasets

# loop through datasets again

for (i2 in datasets){

if (i == i2){

# make and evaluate cross-validation predictions (same dataset)

sc.obj.train <- make.predictions(sc.obj.train)

sc.obj.train <- evaluate.predictions(sc.obj.train)

auroc.all <- auroc.all %>%

add_row(study.train=i, study.test=i,

AUC=eval_data(sc.obj.train)$auroc %>% as.double())

} else {

# make and evaluate on the external datasets

# use meta.ind here, since we want only one sample per subject!

meta.test <- map_combined %>%

filter(dataset_number==i2) %>%

as.data.frame()

rownames(meta.test) <- rownames(meta.test)

sc.obj.test <- siamcat(feat=feat.all, meta=meta.test,

label='STATUS', case='diseased')

# make holdout predictions

sc.obj.test <- make.predictions(sc.obj.train,

siamcat.holdout = sc.obj.test)

sc.obj.test <- evaluate.predictions(sc.obj.test)

auroc.all <- auroc.all %>%

add_row(study.train=i, study.test=i2,

AUC=eval_data(sc.obj.test)$auroc %>% as.double())

}

}

# save the trained model

sc.list[[i]] <- sc.obj.train

}

#After we trained and applied all models, we can calculate the test average for each dataset:

test.average <- auroc.all %>%

filter(study.train!=study.test) %>%

group_by(study.test) %>%

summarise(AUC=mean(AUC), .groups='drop') %>%

mutate(study.train="Average")

#Now that we have the AUROC values, we can plot them into a nice heatmap:

# combine AUROC values with test average

pdf('metaaalysis_RF.pdf',width=7,height=7);

bind_rows(auroc.all, test.average) %>%

# highlight cross validation versus transfer results

mutate(CV=study.train == study.test) %>%

# for facetting later

mutate(split=case_when(study.train=='Average'~'Average', TRUE~'none')) %>%

mutate(split=factor(split, levels = c('none', 'Average'))) %>%

# convert to factor to enforce ordering

mutate(study.train=factor(study.train, levels=c(datasets, 'Average'))) %>%

mutate(study.test=factor(study.test, levels=c(rev(datasets),'Average'))) %>%

ggplot(aes(y=study.test, x=study.train, fill=AUC, size=CV, color=CV)) +

geom_tile() + theme_minimal() +

# text in tiles

geom_text(aes_string(label="format(AUC, digits=2)"),

col='white', size=2)+

# color scheme

scale_fill_gradientn(colours=rev(c('darkgreen','forestgreen',

'chartreuse3','lawngreen',

'yellow')), limits=c(0.5, 1)) +

# axis position/remove boxes/ticks/facet background/etc.

scale_x_discrete(position='top') +

theme(axis.line=element_blank(),

axis.ticks = element_blank(),

axis.text.x.top = element_text(angle=45, hjust=.1),

panel.grid=element_blank(),

panel.border=element_blank(),

strip.background = element_blank(),

strip.text = element_blank()) +

xlab('Training Set') + ylab('Test Set') +

scale_color_manual(values=c('#FFFFFF00', 'grey'), guide=FALSE) +

scale_size_manual(values=c(0, 1), guide=FALSE) +

facet_grid(~split, scales = 'free', space = 'free')

dev.off()

weight.list <- list()

for (d in datasets){

sc.obj.train <- sc.list[[d]]

# extract the feature weights out of the SIAMCAT object

temp <- feature_weights(sc.obj.train)

temp$species <- rownames(temp)

# save selected info in the weight.list

weight.list[[d]] <- temp %>%

select(species, median.rel.weight, mean.rel.weight, percentage) %>%

mutate(Study=d) %>%

mutate(r.med=rank(-abs(median.rel.weight)),

r.mean=rank(-abs(mean.rel.weight)))

}

# combine all feature weights into a single tibble

df.weights <- bind_rows(weight.list)

df.weights <- df.weights %>% filter(grepl('s__', species))

head(df.weights)

strsplit(df.assoc$species, 's__')[2]

df.weights$species <- as.character(map(strsplit(df.weights$species, split = 's__'), 2))

abs.weights <- df.weights %>%

group_by(Study) %>%

summarise(sum.median=sum(abs(median.rel.weight)),

sum.mean=sum(abs(mean.rel.weight)),

.groups='drop')

pdf('InvestigateFeatureWeight_RF.pdf',width=7,height=7);

df.weights %>%

full_join(abs.weights) %>%

# normalize by the absolute model size

mutate(median.rel.weight=median.rel.weight/sum.median) %>%

# only include genera of interest

filter(species %in% genera.of.interest$species) %>%

# highlight feature rank for the top 20 features

mutate(r.med=case_when(r.med > 20~NA_real_, TRUE~r.med)) %>%

# enforce the correct ordering by converting to factors again

mutate(species=factor(species, levels = rev(genera.of.interest$species))) %>%

mutate(Study=factor(Study, levels = datasets)) %>%

ggplot(aes(y=species, x=Study, fill=median.rel.weight)) +

geom_tile() +

scale_fill_gradientn(colours=rev(

c('#007A53', '#009F4D', "#6CC24A", 'white',

"#EFC06E", "#FFA300", '#BE5400')),

limits=c(-0.15, 0.15)) +

theme_minimal() +

geom_text(aes(label=r.med), col='black', size= 2) +

theme(panel.grid = element_blank()) +

xlab('') + ylab('') +

theme(axis.text = element_text(size=6))

dev.off()

# ###METAVOLCANO PROCEDURE

#load files of the 5 datasets lof2Fc based on deseq2

log2fc_1 <- read.delim("dataset1.txt", header=TRUE, sep = "\t")

log2fc_2 <- read.delim("dataset2.txt", header=TRUE, sep = "\t")

log2fc_3 <- read.delim("dataset3.txt", header=TRUE, sep = "\t")

is.numeric(log2fc_1$log2FoldChange)

#build the list datasets

listOfDataframe = list(data1=log2fc_1, data2=log2fc_2,

data3=log2fc_3)

colnames(listOfDataframe$data1)

#combining approach

meta_degs_comb <- combining_mv(diffexp=listOfDataframe,

pcriteria='pvalue',

foldchangecol='log2FoldChange',

genenamecol='PATH',

geneidcol=NULL,

metafc='Mean',

metathr=0.10,

collaps=TRUE,

jobname="MetaVolcano",

outputfolder=".",

draw='HTML')

meta_degs_comb

# Combining results

head(meta_degs_comb@metaresult, 3)

# Plot MetaVolcano

pdf('Metavolcano_plot.pdf', width = 5, height = 5);

meta_degs_comb@MetaVolcano

dev.off()

# ## TEST OTHER DISEASES

#unique ROC T2D

pdf('model_sig_T2D.pdf',width=8,height=8);

pROC_obj <- roc(dif_species_sum$disease, dif_species_sum$risk_index,

smoothed = TRUE,

# arguments for ci

ci=TRUE, ci.alpha=0.9, stratified=FALSE,

# arguments for plot

plot=TRUE, auc.polygon=TRUE, max.auc.polygon=TRUE, grid=TRUE,

print.auc=TRUE, show.thres=TRUE)

dev.off()

sens.ci <- ci.se(pROC_obj)

plot(sens.ci, type="shape", col="lightblue")

## Warning in plot.ci.se(sens.ci, type = "shape", col = "lightblue"): Low

## definition shape.

plot(sens.ci, type="bars")

#unique ROC mucositis

pdf('model_sig_MUC.pdf',width=8,height=8);

pROC_obj <- roc(dif_species_sum_MUC$disease, dif_species_sum_MUC$risk_index,

smoothed = TRUE,

# arguments for ci

ci=TRUE, ci.alpha=0.9, stratified=FALSE,

# arguments for plot

plot=TRUE, auc.polygon=TRUE, max.auc.polygon=TRUE, grid=TRUE,

print.auc=TRUE, show.thres=TRUE)

dev.off()

sens.ci <- ci.se(pROC_obj)

plot(sens.ci, type="shape", col="lightblue")

## Warning in plot.ci.se(sens.ci, type = "shape", col = "lightblue"): Low

## definition shape.

plot(sens.ci, type="bars")

pdf('model_ROC.pdf',width=8,height=8);

ggplot(df, aes(1 - specificity, sensitivity, color = model)) +

geom_textsmooth(aes(label = model), size = 7, se = FALSE, span = 0.2,

textcolour = "black", vjust = 1.5, linewidth = 1,

text_smoothing = 50) +

geom_abline() +

scale_color_brewer(palette = "Set1", guide = "none", direction = 1) +

scale_x_continuous("False Positive Rate", labels = scales::percent) +

scale_y_continuous("True Positive Rate", labels = scales::percent) +

coord_equal(expand = FALSE) +

theme_classic(base_size = 20) +

theme(plot.margin = margin(10, 30, 10, 10))

dev.off()
