# Supplementary material for: Pooled analysis of oral microbiome profiles defines robust signatures associated with periodontitis
Source: mSystems. 2024 Oct 24;9(11):e00930-24. doi: 10.1128/msystems.00930-24 (PMC11575188; doi:10.1128/msystems.00930-24)
Supplement: Legends — for supplemental figures. [file msystems.00930-24-s0006.docx]

**Supplemental Material: Supplemental Figure legends**

**Supplemental Figure S1.** **A)** Principal-coordinate analysis of the matrix of species distances for periodontitis sites and healthy sites, and colored by study. **B)** Principal-coordinate analysis of the matrix of pathways distances for periodontitis sites and healthy sites, and colored by study. **C)** Alpha diversity of periodontitis sites and healthy sites, depicted by species richness, and colored by study. **D)** Alpha diversity of periodontitis sites and healthy sites, depicted by the shannon index, and colored by study. **E)** Alpha diversity of periodontitis sites and healthy sites, depicted by pathways richness, and colored by study. **F)** Alpha diversity of periodontitis sites and healthy sites, depicted by the Shannon index at pathways level, and colored by study.

**Supplemental Figure S2. A)** Most common dominant bacteria in the oral microbiome in the Shi et al study. **B)** Most common dominant bacteria in the oral microbiome in the newly sequenced metagenomes (Soueidan et al study). **C)** Triad of the Socransky’s Red Complex in the Shi et al study. **D)** Triad of the Socransky’s Red Complex in the newly sequenced metagenomes (Soueidan et al study).

**Supplemental Figure S3. A)** Prediction matrix for microbiome-based prediction of diet within each dataset (values on the diagonal), across pairs of cohorts (one cohort used to train the model and the other for testing). We report the AUC-ROC values obtained from Random Forrest models on species-level relative abundances. Values on the diagonal refer to the median AUC-ROC values of 100-repeated fivefold-stratified cross-validations. Off-diagonal values refer to AUC-ROC values obtained by training the classifier on the cohort of the corresponding row and applying it to the cohort of the corresponding column. **B)** The same prediction matrix using functional microbiome profiles (at pathways level).

**Supplemental Figure S4. A)** Fast-and-frugal tree-based staging scheme to predict periodontitis in a train dataset at species level. **B)** Fast-and-frugal tree-based staging scheme to predict periodontitis in a test dataset at species level. **C)** Fast-and-frugal tree-based staging scheme to predict periodontitis in a train dataset at species level. **D)** Fast-and-frugal tree-based staging scheme to predict periodontitis in a test dataset at species level.
